# A Cross-sectional Study of *KLKB1* and *PRCP* Polymorphisms in Patient Samples with Cardiovascular Disease

**DOI:** 10.3389/fmed.2016.00017

**Published:** 2016-04-29

**Authors:** Haley R. Gittleman, Alona Merkulova, Omar Alhalabi, Evi X. Stavrou, Martina L. Veigl, Jill S. Barnholtz-Sloan, Alvin H. Schmaier

**Affiliations:** ^1^Case Comprehensive Cancer Center, Case Western Reserve University, Cleveland, OH, USA; ^2^Department of Medicine, Division of Hematology-Oncology, Case Western Reserve University, Cleveland, OH, USA; ^3^Louis Stokes Veterans Administration Hospital, Cleveland, OH, USA; ^4^University Hospitals Case Medical Center, Cleveland, OH, USA

**Keywords:** cardiovascular disease, prekallikrein, *KLKB1*, prolylcarboxypeptidase, *PRCP*, high molecular weight kininogen

## Abstract

Plasma kallikrein formed from prekallikrein (PK) produces bradykinin from kininogens and activates factor XII. Plasma PK is activated by factors αXIIa, βXIIa, or prolylcarboxypeptidase (PRCP). A cross-sectional investigation determined if there is an association of *PRCP* and *KLKB1* polymorphisms with cardiovascular disease (CVD). DNA was obtained from 2243 individuals from the Prevention of Events with Angiotensin Converting Enzyme trial. Two *PRCP* SNPs, rs7104980 and rs2298668, and two *KLKB1* SNPs, rs3733402 and rs3087505, were genotyped. Logistic regression models were performed for history of diabetes, myocardial infarction, stroke, angina, angiographic coronary disease, CABG, intermittent claudication, percutaneous transluminal coronary angioplasty (PTCA), and transient ischemic attack. The *PRCP* SNP rs7104980 increased the odds of having a history of PTCA by 21% [odds ratio (OR) = 1.211; 95% confidence intervals (CI) = (1.008, 1.454)]; *P* = 0.041, but was non-significant after Bonferroni correction. Alternatively, having the G allele for rs3733402 (*KLKB1* gene) decreased the odds of having a history of angiographic coronary disease by 24% [OR = 0.759; 95% CI = (0.622, 0.927)]; *P* = 0.007 that was statistically significant (*P* < 0.01) after Bonferroni correction for multiple hypothesis testing. When the best-fit model based on the Akaike information criterion controlled for age, weight, gender, hypertension, and history of angina, the G allele of *KLKB1* rs3733402 that is associated with less plasma kallikrein activity correlated with reduced history of CVD.

## Introduction

Plasma prekallikrein (PK) is a zymogen whose enzyme plasma kallikrein is known to initiate and amplify factor XII activation on collagen and polyphosphates in plasma in the intravascular compartment to activate intrinsic blood coagulation. Formed plasma kallikrein is also the major enzyme to generate bradykinin from high molecular weight kininogen (HK) and to convert plasminogen to plasmin and single-chain urokinase to two-chain urokinase for fibrinolysis, C3 and C5 to C3a and C5a, respectively, in the complement system and prorenin to renin in the renin angiotensin system. There are several SNPs that have been recognized to impact PK function. *KLKB1* rs3087505 is associated with venous thrombosis and this SNP is in linkage disequilibrium (LD) with two *F11* SNPs (rs2036914, rs3756008) also associated with thromboembolism (1, 2). Also an exon 5 N124S polymorphism (rs3733402) in Apple domain 2 results in reduced PK complex formation to HK, its plasma, and cell membrane-binding protein (3–5), and this interferes with optimal PK activation and BK formation. Recent studies with *klkb1^−/−^* mice indicate that animals lacking *klkb1* have delayed arterial thrombosis not due to reduced contact activation but due to reduced vessel wall tissue factor (6, 7). These combined studies suggest that PK contributes to vascular homeostasis. Infact, previous work by our own group demonstrated that elevated plasma PK correlates with accelerated vascular disease and proteinuria in a large population of diabetic patients (8, 9).

Prolylcarboxypeptidase (PRCP) is a membrane-associated serine protease that cleaves peptides where the penultimate position is a Pro-X bond (10, 11). As an exopeptidase, it has been recognized to degrade desArg9-bradykinin, angiotensin II and III, and α-melanocyte-stimulating hormone_1–13_ (10–12). We also have shown that it is a plasma PK activator when PK assembles on HK on endothelial and other cell membranes, suggesting that it is an endopeptidase as well (13–15). Genetic studies have identified an association between *PRCP* and essential hypertension and metabolic syndrome (16, 17). An exon SNP of *PRCP* (rs2298668, E112D) indicates risk for chronic hypertension and preeclampsia in an African-American patient population (18). In a Han Chinese population, it is associated with treatment resistance to an ACE inhibitor (19). In another study of Han Chinese subjects, the G allele of intron SNP rs7104980, but not rs2298668, is associated with essential hypertension [odds ratio (OR) = 1.98, 95% confidence intervals (CI) = 1.62–2.43, *P* = 0.3 × 10^−10^] (20). PRCP-deficient gene-trap (*prcp^*gt/gt*^*) mice that have 25% PRCP normal protein levels in their organs are constitutively hypertensive and have a heightened risk for arterial thrombosis (21). Likewise, spontaneous hypertensive rats (SHR) compared to their control Wistar rats also have reduced PRCP in cardiac tissue (22). PRCP deficiency in mice is associated with increased vascular inflammation and reduced cell proliferation, angiogenesis, and wound repair from physical injury and ischemia reperfusion (23). Opposite to PK, the presence of PRCP reduces vascular inflammation and arterial thrombosis.

In the present investigation, we asked if polymorphisms of PRCP and PK are associated with cardiovascular disease (CVD). In a single cross-sectional study, we show that the G allele for *PRCP* intronic rs7014980 associates with CVD but does not meet statistical significance. Alternatively, the minor G allele of an exonic SNP rs3733402 (N124S) of *KLKB1* is significantly associated with reduced CVD. These data suggest that a selected *KLKB1* SNP (rs3733402) mirrors the phenotype observed in patient studies with diabetes and on murine deletions of *klkb1* (7–9).

## Materials and Methods

### Data Collection

Plasma samples for our cross-sectional investigation below were provided from the Prevention of Events with Angiotensin Converting Enzyme (PEACE) Inhibition Trial that were in the NIH Biorepository BioLincc (https://biolincc.nhlbi.nih.gov/home/) (24). In the original PEACE trial, all participants signed informed consent that was approved by their local IRBs for blood collection (24). The goal of the PEACE trial was to test whether ACE-inhibitor therapy would reduce the rate of non-fatal myocardial infarction (MI), death from cardiovascular causes, or coronary revascularization in low-risk patients with stable coronary artery disease and normal or slightly reduced left ventricular function (ejection fraction >40%). The criteria for selection in the PEACE trial were individuals >50 years old with evidence of coronary artery disease documented by one of the following three criteria: (1) MI >3 months from enrollment, (2) coronary artery bypass grafting (CABG) or percutaneous transluminal coronary angioplasty (PTCA) >3months from enrollment, and (3) obstruction of at least one luminal diameter >50% of at least one native vessel on coronary angiography, and toleration of the medication and >80% compliance with the medication (24). The cardiovascular phenotypes were defined in the PEACE trial and obtained from its data dictionary (24). The outcomes of the present analysis were compared with the baseline, pretreatment cardiovascular phenotype of the subjects. We determined if the present selected SNPs correlated with the mild cardiovascular phenotypes of the subjects of the PEACE trial.

The subsample of subject plasmas from the repository were de-identified from the individual donors but were linked by code to clinical information in the data dictionary (23). The samples that were provided by NHLBI BioLincc were chosen by random availability of 1 ml plasma aliquots for shipment. We obtained an exempt approval from the University Hospitals Case Medical Center IRB NHR-12-13. Two thousand two hundred forty-three (*N* = 2243) plasma samples from the PEACE trial were used for DNA isolation. DNA was extracted by the NucleoSpin Plasma XS technique (Macherey-Nagel, Inc.) yielding 0.03–0.1 μg/sample. The extracted DNA had whole-genome amplification using REPLI-g Mini Kit (Cat# 150023) (QIAGEN) to yield 10 mg/ml. DNA samples were quantified using Qubit DNA quantification assay and a Qubit 2.0 Fluorometer (Life Technologies).

As described above, two SNPs from *PRCP* that have been associated with hypertension, intronic rs7104980 (11:82864153) (20) and exonic rs2298668 (11:82853252) (E112D) (18, 19) were studied. Also, two SNPs from *KLKB1* that have been associated with vascular function were chosen for study; rs3087505 (Chromosome location, 4:186258332) (1, 2) from the 3′UT and exonic rs3733402 (4:186236880) (N124S) (3). All SNPs studied were listed as Illumina Golden Gate validated and ABI TaqMan genotyping assays were available for them. ABI TaqMan chemistry consists of two probes labeled with either VIC or FAM and a primer pair to detect the specific SNP. Genotyping these samples was performed according to the manufacturer’s protocol. Specifically, 3–5 μl aliquots, containing 5–10 ng of DNA were transferred from 96-well reservoir plates to 384-well assay plates for each individual being genotyped. Multiple 384-well assay plates were generated; the DNA was dried down, the plates then sealed, and frozen until assayed. A 5 μl aliquot of Master Mix, Probe and Primer was robotically added to each well of a 384-well plate previously plated with DNA. PCR (40 cycles) was carried out on an ABI GeneAmp PCR System 9700 Dual Head Instrument and endpoint reads were carried out using the ABI 7900 Sequence Detection System (SDS). All assay mixtures were prepared in an amplicon-free room to avoid contamination. Data from each SNP were clustered using ABI’s SDS version 2.3 Software and Genotyping Calls (Vic/Vic; Fam/Fam; Vic/Fam) were automatically made by the software. Each file was manually inspected to remove outliers. The error rate of each SNP not identifying the genotype of eight control DNA samples examined 15 times over the course of the study was 6.9% for rs7104980, 6.2% for rs3087505, 13% for rs2298668, and 7.4% for rs3733402. These rates are similar to other large-scale genetic epidemiology studies.

### Statistical Analysis

Genotyping rates were calculated for each SNP across all individuals. Allele and genotype frequencies were calculated for each SNP. Hardy–Weinberg equilibrium (HWE) and pairwise LD were assessed using standard methods. A heatmap was used to determine if SNP rs3733402 is in LD with *F11* SNPs rs2036914 and rs3756008, respectively. Unconditional logistic regression models assessed the association between candidate SNPs and various disease outcomes calculating ORs and 95% CI. For each candidate SNP, having the rarer allele was compared to not having the rarer allele. Other covariates included in the logistic regression models were age, weight, gender, hypertension status, history of diabetes, history of angina, and/or cigarette use. Hypertension in the PEACE trial was defined as having systolic blood pressure greater or equal to 140 mmHg and/or having diastolic blood pressure greater or equal to 90 mmHg (24). This variable is user-defined specific for this investigation and different from the history of hypertension variable used in the PEACE trial. For cigarette use, the “rarely” and “never” categories were combined and compared against “frequent” cigarette use. Logistic regression models were then compared using specific −2 log likelihood values in order to calculate the Akaike information criterion (AIC) (25). The result with the smallest AIC was chosen as the best-fitting model. Multiple hypothesis testing was corrected by the Bonferroni correction method. All statistical analyses were performed using SAS version 9.3 and R.

## Results

### Frequencies

Patient characteristics are presented in Table 1. Ninety-two percent of the subjects in the control and treated groups of the PEACE trial were Caucasians. The mean age of the 2243 successfully genotyped participants was 64 ± 8 years, female (18%), frequently smoked cigarettes (14%), and had a history of transient ischemic attack (TIA) (3%), stroke (4%), intermittent claudication (8%),  diabetes (15%), and CABG (36%). The majority of patients did have a history of angina (71%) and angiographic coronary disease (66%). These patient characteristics were similar to the overall PEACE study population (24). This subset study that represents 2243 out of the total 8290 patients in the overall PEACE study population (27% of total) had similar clinical characteristics as the PEACE trial with exception of the incidence of hypertension (current trial 25 vs. 45% in PEACE), history of PTCA (current trial 43 vs. 72% in PEACE), and history of MI (current trial 42 vs. 55% in PEACE) (24).

**Table 1:** **Individual characteristics of the studied population (*N* = 2243 from the PEACE trial^b^)**.

Variable	Level	Frequency	Column percentage
Relative age	Under 51	58	2.59
	51–55	364	16.23
	56–60	414	18.46
	61–65	440	19.62
	66–70	485	21.62
	71–75	305	13.60
	76–80	155	6.91
	Over 80	22	0.98
Sex	Male	1846	82.30
	Female	397	17.70
Hypertension^a^	No	1661	74.05
	Yes	582	25.95
Cigarette use	Frequently	313	13.96
	Rarely	1406	62.71
	Never	523	23.33
History of diabetes	No	1914	85.33
	Yes	329	14.67
History of myocardial infarction	No	935	41.70
	Yes	1307	58.30
History of stroke	No	2152	95.99
	Yes	90	4.01
History of angina	No	658	29.35
	Yes	1584	70.65
History of angiographic coronary disease^c^	No	770	34.33
	Yes	1473	65.67
History of CABG	No	1435	63.98
	Yes	808	36.02
History of intermittent claudication	No	2061	91.89
	Yes	182	8.11
History of PTCA	No	1263	56.33
	Yes	979	43.67
History of TIA	No	2170	96.75
	Yes	73	3.25

*^a^Hypertension was defined as having systolic blood pressure greater or equal to 140 mmHg and/or having diastolic blood pressure greater or equal to 90 mmHg (24)*.

*^b^The larger PEACE trial was comprised of 8290 patients; this study represents 27% of the overall total*.

*^c^History of angiographic disease means documented by coronary angiography*.

Allele and genotype frequencies are presented in Table 2 for the candidate SNPs in the *PRCP* and *KLKB1* genes. Across all four SNPs, the frequency of undetermined genotypes ranged from 5.1 to 8.4%. For rs7104980 in the *PRCP* gene, the less common allele was G (44.7%) and the rarer genotype was the G homozygote (G/G) (29%). For rs2298668 in the *PRCP* gene, the less common allele was the G allele (8.3%) and the rarer genotype was the G homozygote (G/G) (5.8%). For rs3087505 in the *KLKB1* gene, the less common allele was the A allele (10.29%) and the rarer genotype was the A homozygote (A/A) (3.3%). For rs3733402 in the *KLKB1* gene, the less common allele was the G allele (48.1%) and the rarer genotype was G/G (31.4%).

**Table 2:** **Allele and genotype frequencies for the *PRCP* and *KLKB1* SNPs**.

**Allele frequencies**						
Gene	SNP	Allele	Count	Frequency	dbSNP Hapmap CEU frequency	
*PRCP*	rs7104980	C	2197	0.525	0.553	
		G	1985	0.475	0.447	
	rs2298668	G	684	0.165	0.083	
		T	3460	0.835	0.917	
*KLKB1*	rs3733402	A	2080	0.506	0.519	
		G	2028	0.494	0.481	
	rs3087505	A	438	0.103	0.088	
		G	3820	0.897	0.912	
**Genotype frequencies**						
**Gene**	**SNP**	**Genotype**	**Count**	**Frequency**	**dbSNP Hapmap CEU frequency**	

*PRCP*	rs7104980	C/C	713	0.341	0.301	
		C/G	771	0.369	0.504	
		G/G	607	0.290	0.195	
	rs2298668	G/G	120	0.058	0.017	
		G/T	444	0.214	0.133	
		T/T	1508	0.728	0.850	
*KLKB1*	rs3733402	A/A	670	0.326	0.302	
		A/G	740	0.360	0.434	
		G/G	644	0.314	0.264	
	rs3087505	A/A	70	0.033	0.018	
		A/G	298	0.140	0.142	
		G/G	1761	0.827	0.841	
**Hardy–weinberg equilibrium (HWE) and linkage disequilibrium (LD) measures**						
**Gene**	**Test for HWE**			**Linkage disequilibrium measures**		
	**SNP**	**Chi-square**	***P*-value**	**Locus 1**	**Locus 2**	**Correlation coefficient**

*PRCP*	rs7104980	142.0634	9.999 × 10^−5^	rs7104980	rs2298668	0.372
	rs2298668	102.6263	9.999 × 10^−5^			
*KLKB1*	rs3733402	160.2745	9.999 × 10^−5^	rs3733402	rs3087505	0.277
	rs3087505	124.2959	9.999 × 10^−5^			

### Assessment of Hardy–Weinberg Equilibrium and Linkage Disequilibrium

**Figure 1:**
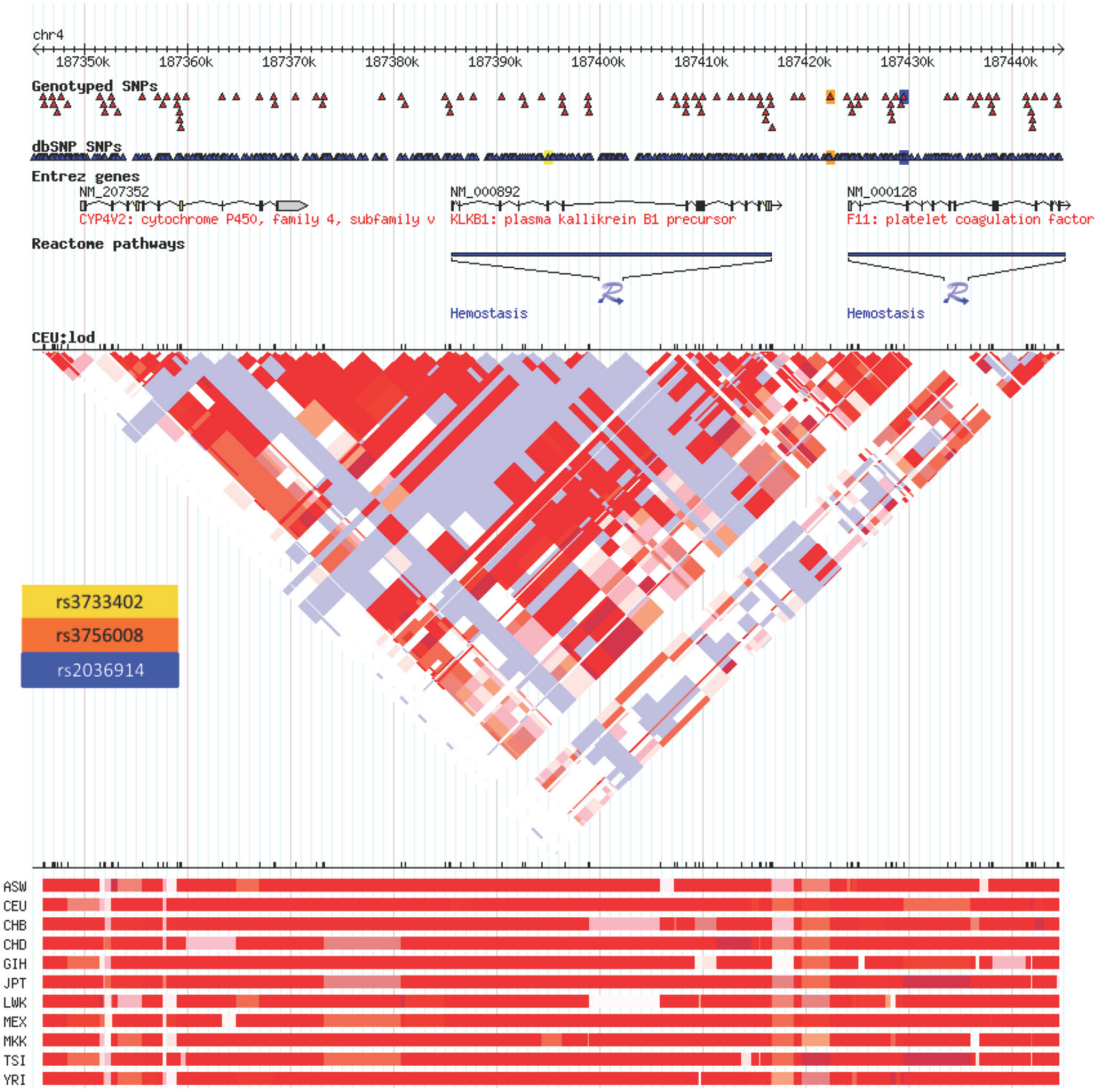
**Linkage disequilibrium heatmap for rs3733402 vs. F11 SNPs**. *KLKB1* SNP rs3733402 is not in LD with *F11* SNPs rs2036914 and rs3756008, although the two F11 SNPs are in LD with each other. The LD heatmap is gray between the KLKB1 and F11 genes and red within the F11 gene.

The Hardy–Weinberg chi-square values for each SNP (HWE), as well as the LD correlation coefficients between each SNP per gene, were computed. Low *P*-values associated with the chi-square statistic for the HWE tests were observed that were the result of a large sample size, suggesting that the SNPs were in violation of HWE. This finding was due to having a statistical power large enough to detect small differences in this study that were not truly biologically meaningful. Hence, no SNPs were removed. In addition, both the allele and genotype frequencies for all tested SNPs were very similar to those from the dbSNP Hapmap CEU frequencies (Table 2). There were no SNPs in significant LD. The correlation coefficient between the two *PRCP* SNPs was 0.372, and the correlation coefficient between the two *KLKB1* SNPs was 0.277, which were both smaller than the cutoff value of 0.8 for assessing significant LD. In a previous study examining SNPs associated with venous thrombosis, *KLKB1* SNP rs3087505 was found to be in LD with two *F11* SNPs rs2036914 and rs3756008 (1). We determined that *KLKB1* SNP rs3733402 is not in LD with *F11* SNPs rs2036914 and rs3756008, although the two F11 SNPs are in LD with each other (Figure 1).

### Logistic Regression Models and Model Selection

Initially, logistic regression models were run for various CVD-associated outcomes, including history of diabetes, MI, stroke, angina, angiographic coronary disease, CABG, intermittent claudication, PTCA, and TIA. The “baseline” model controlled for patient age, weight, and gender (Table 3). No associations were found for the *PRCP* rs2298668 and *KLKB1* rs3087505 SNPs. Having the rare G allele compared to not having the G allele for rs7104980 (PRCP gene) increased the odds of having a history of PTCA by 21% [OR = 1.211; 95% CI = (1.008, 1.454); *P* = 0.041]. Alternatively, having the rarer G allele compared to not having the G allele for rs3733402 (*KLKB1* gene) decreased the odds of having a history of angiographic coronary disease by 24% [OR = 0.759; 95% CI = (0.622, 0.927); *P* = 0.007]. After correcting for testing multiple SNPs by the Bonferroni correction method, only SNP rs3733402 (*KLKB1* gene) was significantly associated with an outcome of having a history of angiographic coronary disease (adjusted *P* < 0.0125) (Table 3).

**Table 3:** **Association of cardiovascular disease outcomes with SNPs in the *PRCP* and *KLKB1* genes controlling for age, weight, and gender**.

Outcome	PRCP						KLKB1					
	rs7104980			rs2298668			rs3733402			rs3087505		
	OR	95% CI	*P*-value	OR	95% CI	*P*-value	OR	95% CI	*P*-value	OR	95% CI	*P*-value
History of diabetes	0.934	(0.723, 1.207)	0.601	0.893	(0.675, 1.183)	0.432	1.054	(0.808, 1.376)	0.696	0.955	(0.689, 1.323)	0.782
History of MI	1.203	(0.998, 1.449)	0.052	1.036	(0.851, 1.263)	0.723	0.958	(0.793, 1.156)	0.652	1.098	(0.874, 1.381)	0.422
History of stroke	1.111	(0.713, 1.732)	0.642	1.013	(0.618, 1.660)	0.959	0.993	(0.623, 1.582)	0.975	0.856	(0.492, 1.487)	0.580
History of angina	0.957	(0.786, 1.166)	0.664	1.068	(0.864, 1.320)	0.541	1.054	(0.859, 1.292)	0.614	0.796	(0.617, 1.029)	0.081
History of angiographic coronary disease	1.062	(0.877, 1.286)	0.535	1.042	(0.848, 1.279)	0.698	**0.759**	**(0.622, 0.927)**	0.007*	0.847	(0.670, 1.071)	0.166
History of CABG	1.001	(0.828, 1.211)	0.988	1.064	(0.869, 1.303)	0.548	0.917	(0.756, 1.113)	0.381	1.076	(0.850, 1.361)	0.542
History of intermittent claudication	0.882	(0.636, 1.225)	0.454	1.135	(0.804, 1.604)	0.471	0.994	(0.710, 1.392)	0.971	0.888	(0.579, 1.362)	0.586
History of PTCA	*1.211*	*(1.008, 1.454)*	*0.041*	1.018	(0.836, 1.239)	0.860	1.094	(0.907, 1.319)	0.347	0.970	(0.772, 1.218)	0.792
History of TIA	0.691	(0.425, 1.125)	0.137	0.638	(0.345, 1.181)	0.153	0.847	(0.506, 1.419)	0.528	1.022	(0.542, 1.928)	0.946

*SNP rs7104980: G allele compared to no G allele*.

*SNP rs2298668: G allele compared to no G allele*.

*SNP rs3733402: G allele compared to no G allele*.

*SNP rs3087505: A allele compared to no A allele*.

*Significant at the Bonferroni corrected *P*-value correction for assessing four total SNPs = 0.05/4 = 0.0125.

In order to better understand the potential association between having the G alleles for rs7104980 and rs3733402 and CVD, we calculated the −2 log-likelihood values in order to calculate the AIC to determine the best-fitting model (25). As shown in Table 4, the final model chosen for rs7104980, which had the lowest AIC, controlled for the baseline covariates (age, gender, and weight), hypertension, and history of angina. Having the G allele compared to not having the G allele for rs7014980 (*PRCP* gene) increased the odds of having hypertension and a history of angina by 22% [OR = 1.220; 95% CI = (1.016, 1.467)]; *P* = 0.034, but this analysis was not statistically significant (*P* > 0.01) after Bonferroni correction. Alternatively, having the G allele compared to not having the G allele for rs3733402 (*KLKB1* gene) decreased the odds of the baseline covariates, hypertension, and history of angina by 24% [OR = 0.762; 95% CI = (0.623, 0.931); *P* = 0.008], which was statistically significant (*P* ≤ 0.01) for multiple testing after Bonferroni correction.

**Table 4:** **Model selection for the best fit for the association between SNP rs7104980 (PRCP gene) and SNP rs3733402 (KLKB1 gene) with hypertension and a history of diabetes, cigarette use, or angina**.

Covariate(s) added	PRCP – rs7104980					KLKB1 – rs3733402				

	History of PTCA					History of angiographic coronary disease				

	OR	95% CI	*P*-value	−2 log L	AIC	OR	95% CI	*P*-Value	−2 log L	AIC
Baseline^a^	1.211	(1.008, 1.454)	0.041	2814.923	2849.357	0.759	(0.622, 0.927)	0.007*	2605.204	2605.204
Baseline^a^ + Hypertension	1.209	(1.007, 1.452)	0.042	2812.164	2847.677	0.761	(0.624, 0.930)	0.007*	2602.004	2604.004
Baseline^a^ + Hypertension + Hx of diabetes	1.208	(1.006, 1.451)	0.043	2807.581	2847.455	0.760	(0.623, 0.928)	0.007*	2600.974	2604.974
Baseline^a^ + Hypertension + Cigarette use	1.214	(1.011, 1.459)	0.038	2804.401	2845.804	0.763	(0.625, 0.931)	0.008*	2600.346	2604.346
Baseline^a^ + Hypertension + Hx of angina	1.220	(1.016, 1.467)	0.034	2744.022	2720.286	0.762	(0.623, 0.931)	0.008*	2573.113	2577.113^b^

*^a^Baseline model includes age, weight, and gender covariates only*.

*^b^Lowest AIC value*.

*Significant at the Bonferroni corrected *P*-value for assessing 5 total models = 0.05/5 = 0.01.

## Discussion

The present cross-sectional study suggests that a polymorphism in *KLKB1* associated with reduced PK binding to HK leading to a less actable zymogen is associated with reduced CVD. However, we are cautious in over interpreting the results of this investigation because it has several limitations. The PEACE trial was performed on low-risk individuals with CVD who had stable coronary artery disease and normal or slightly reduced left ventricular function. The patient population was 82% male with the majority of patients between 51 and 70 years of age with only 14% frequent smokers. The majority of patients did not have TIA, stroke, claudication, diabetes, hypertension, CABG, or PTCA. The present subset analysis of samples from the PEACE trial was similar in most ways, but actually had subjects with significantly reduced incidence of hypertension, PTCA, and MI. Since these three indicators are important disease markers of CVD, their reduction may have influenced the outcomes of the present studies. Overall, the present study examined a healthier subset of individuals with lower risk CVD than in the original PEACE trial itself.

Although there were no associations with the non-coding SNP associated with venous thrombosis, it is of interest that the *KLKB1* exonic SNP, rs3733402 (N124S), was shown to be protective in the studied subjects for having a history of angiographic coronary artery disease (Table 3). Rs3733402 is not in LD with two *F11* SNPs associated with venous thrombosis even though *KLKB1* SNP rs3087505 from the 3′prime UT region is (1). The meaning of SNPs of different genes being in LD and associated with one disorder, such as venous thrombosis, is not known. The N124S polymorphism is in the Apple domain 2 of PK where HK binds (3, 26). Since the majority of plasma PK circulates in complex with HK and HK is a PK receptor on endothelial cells, the polymorphism of rs3733402 may be functionally important *in vivo* (4, 5). Rs3733402, a common polymorphism, is associated with reduced PK binding to HK (3). The polymorphism alters a PK glycosylation site that reduces its ability to bind to HK (3, 26). PK’s ability to bind to HK is important to protect plasma kallikrein from inhibition by C1 inhibitor and for PK’s activation by PRCP on cells (5, 27). This polymorphism may promote C1 inhibition of plasma kallikrein and reduce PRCP activation of PK to plasma kallikrein, thus limiting plasma kallikrein activity in two ways *in vivo* (27, 28).

Previous studies by us have shown that elevated plasma PK is associated with accelerated diabetic vascular disease (8, 9). Elevations of plasma kallikrein may be associated with increased prorenin conversion to renin and renin formation leading to elevated angiotensin II. Recent genome-wide meta-analysis studies indicate that the *KLKB1* SNP rs3733402 has a positive correlation with circulating B-type natriuretic peptide, a recognized cardioprotective vasodilator in African Americans (29). Other recent studies show that PK deficient mice (*klkb1^−/−^* mice) have reduced arterial thrombosis providing support in mouse models to enhance the observation made in human association studies (6–9). It is of interest that mechanism for delayed thrombosis in *klkb1^−/−^* mice is mediated in part through elevated prostacyclin and vasculo-protective transcription factors Sirt1 and KLF4 with reduction of vessel wall tissue factor and not reduced contact activation as is commonly believed (7). The results of the present investigation showing that rs3733402 is associated with decreased CVD are consistent with these other studies (8, 9). Combined these investigations suggest that lowered PK may improve vascular health indirectly by reducing vessel thrombosis risk.

In the present investigation, it might be construed that significance of rs3733402 with a history of angiographic coronary disease is not consistent with the results observed for intermittent claudication, PTCA, and angina (Table 3). The lack of association with intermittent claudication is not surprising because there was a very small group of individuals in the study population with this clinical problem. In a similar manner, one may argue that the population of study subjects with PTCA was also a smaller group than the angiographic coronary disease group. When a logistic regression study was performed with a baseline model, including age, weight, gender, hypertension, and history of angina, history of angina did contribute to the best-fitting model (25) (Table 4).

The studies on *PRCP* SNPs were performed because PK is a substrate of PRCP. Furthermore, investigations show that an absence of PRCP in gene-trap mice is associated with CVD manifested as vascular inflammation, including increased vessel leukocytosis, reactive oxygen species, and apoptosis with reduced cellular proliferation, constitutive anticoagulation, and vessel angiogenesis and repair after mechanical and ischemia/reperfusion injury (21, 23). The current study suggests that the G allele of rs7104980 of *PRCP* may be a cardiovascular risk factor for PTCA, controlling for age, weight, and gender (*P* < 0.041), but the Bonferroni correction of the *P*-value for the total number of SNPs (*P* < 0.0125) shows non-significance (Table 3). Likewise the use of the AIC to choose the best-fitting result did not identify additional subsets such as hypertension or history of angina that increased the significance of this association (Table 4). In this population of patient with less severe CVD, we did not observe *PRCP* SNPs as risk factors. Our investigations were consistent with the investigations of Wu et al. who showed that the G allele of SNP rs710980 was associated with susceptibility for essential hypertension in 1020 Han Chinese (20). However, our present study has results that do not meet the significance level set by our investigations’ parameters. The implication of these data may be that polymorphisms in PRCP may be significant in patients with more serious hypertensive CVD than those investigated in the present study.

Furthermore, our studies like others did not show any significant association with the rs2298668 *PRCP* polymorphism (E112D PRCP) that was associated with chronic hypertension with or without increased risk of preeclampsia or resistance to an ACE-inhibitor therapy for hypertension (18, 19). First, the studies of Wang et al. and Zhang et al. were with patients who had more advanced hypertension and signs of deteriorating cardiovascular health than the subjects examined in the present report (18, 19). This difference also accounts for the different results with SNP rs2298668 between these two studies and the one of Wu et al. (18–20). The investigation of Wu et al. used subjects who just had essential hypertension without history of diabetes mellitus, MI, cerebrovascular accident, or other serious diseases (20). Finally, since previous studies with SNPs rs7104980 and rs2298668 were performed on Asian and African-Americans subjects, respectively, the non-statistically significant results observed in the present study also may be related to the mostly male Caucasian population examined.

When we performed this investigation, we were concerned whether we were able to get adequate DNA from stored plasma samples. We avoided serum samples for DNA preparation in this investigation since it has been associated with degraded DNA (30). We reasoned that well-collected plasma samples properly stored preserves soluble DNA for adequate preparation for assay like it does for study of blood coagulation zymogens. Our studies show that the genotyping error rate of 5–8.4% in the DNA prepared from subject plasma samples is less than that of 6.2–13% error rate in control DNA prepared from tissue across the SNPs.

Another caution in interpreting these findings is that this investigation is based only on a single cross-sectional study of samples. It needs to be confirmed by similar investigations on additional sample sets. Furthermore, the results of this cross-sectional study should be confirmed in a case-controlled study to obviate any unintended selection bias.

In sum, we postulate that the G allele of a *KLKB1* SNP, rs3733402, is associated with reduced hypertension and coronary artery disease. These data suggest that reduction of PK activity may reduce CVD consistent with our previous studies in diabetic patients (8, 9). Our current findings also are consistent with our murine models. In our animal models, PK and PRCP may have opposing activities in the cardiovascular system regulating our risks for arterial disease. Reduction of PK or elevation of PRCP may be future targets to promote vascular health to reduce CVD.

## Conclusion

This paper is the first cross-sectional investigation on two *KLKB1* SNPs on a group of patients with mild CVD. When subject age, male gender, and weight are taken into account, *KLKB1* SNP rs3733402 significantly associates with those with a reduced history of angiographic coronary artery disease. When subjects have covariates of age, weight, gender, hypertension, rs3733402 best associates with those individuals who both have a reduced history of angina. Rs3733402, an exonic SNP that is associated with reduced PK activation, also is associated with reduced CVD.

## Author Contributions

JB-S and AS designed the project. AM, OA, MV, and ES performed experimental work. HG, JB-S, MV, and AS performed data analysis. HG, JB-S, MV, ES, and AS performed manuscript drafting and editing.

## Conflict of Interest Statement

The authors declare that the research was conducted in the absence of any commercial or financial relationships that could be construed as a potential conflict of interest.

## Abbreviations

AIC, akaike information criterion; CVD, cardiovascular disease; *KLKB1*, plasma prekallikrein gene; PEACE, prevention of Events with Angiotensin Converting Enzyme Inhibition Trial; PK, plasma prekallikrein protein; *PRCP*, prolylcarboxypeptidase gene; PRCP, prolylcarboxypeptidase protein.
